# New insights on poly(*cis*-1,4-isoprene) rubber degradation through enzymatic kinetics and process improvement

**DOI:** 10.3389/fbioe.2025.1593339

**Published:** 2025-05-02

**Authors:** Camila Guajardo-Flores, Josefa Rojas, Yvan Baldera-Moreno, Francisco Adasme-Carreño, Daisuke Kasai, Rodrigo Andler

**Affiliations:** ^1^ Facultad de Ciencias de la Ingeniería, Universidad Católica del Maule, Talca, Chile; ^2^ Escuela de Ingeniería en Biotecnología, Centro de Biotecnología de los Recursos Naturales (CenBio), Universidad Católica del Maule, Talca, Chile; ^3^ Departamento de Ciencias Pre-Clínicas, Facultad de Medicina, Universidad Católica del Maule, Talca, Chile; ^4^ Centro de Investigación de Estudios Avanzados del Maule, Vicerrectoría de Investigación y Postgrado, Universidad Católica del Maule, Talca, Chile; ^5^ Laboratorio de Bioinformática y Química Computacional, Departamento de Medicina Traslacional, Facultad de Medicina, Universidad Católica del Maule, Talca, Chile; ^6^ Department of Materials Science and Bioengineering, Nagaoka University of Technology, Nagaoka, Niigata, Japan

**Keywords:** biocatalysis, enzymatic degradation, latex clearing protein, poly(*cis*-1,4-isoprene), rubber biodegradation, oligoisoprenoids

## Abstract

Latex clearing protein (Lcp) is a crucial enzyme in the oxidative degradation of poly(*cis*-1,4-isoprene), the main component of natural rubber (NR). Despite significant biochemical advances, to date, the kinetic behavior of Lcp from *Streptomyces* sp. K30 (Lcp_K30_) has not been characterized, limiting the efficiency of NR conversion. In this work, Lcp_K30_ was produced in *Escherichia coli* BL21 (DE3) + p4782.1::*lcp*_K30_ with L-rhamnose as the inducer, yielding 6.05 mg/L of purified protein. Kinetic assays demonstrated a positive correlation between the initial reaction rate and poly(*cis*-1,4-isoprene) concentration, reaching a maximum rate of 7.05 nmol O_2_/min at the enzyme’s saturation point, corresponding to 5 μg Lcp/mg NR. The Michaelis–Menten constant (K_m_) was determined to be 308.3 mg/mL, with the Hill model providing the best fit for the data. NR-to-oligoisoprenoid conversion reached 12.9 mg in 24 h, exceeding previously reported yields, while gel permeation chromatography analysis indicated conversion efficiencies over 80%, far exceeding the reports of previous studies where only 30%–40% conversions were achieved. Furthermore, Fukui function analysis suggested that the aldehyde terminal groups of the oligoisoprenoids may be less susceptible to enzymatic degradation, which would explain the mass distribution of the degradation products.

## Graphical Abstract

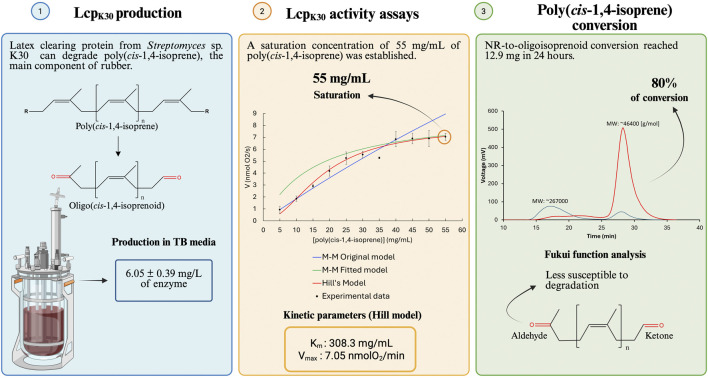

## 1 Introduction

Natural rubber (NR) is a polymer that possesses unique physical properties and has been widely used by mankind for more than a century. As a result of modern life, the use of natural or synthetic rubber-based products, such as tires, insulation, and waterproofing products, among others, has increased (Leong et al., 2023). This leads to problems in waste management once these materials reach the end of their useful life. More than 25 million metric tons of natural and synthetic rubber are produced and consumed annually, causing problems such as (i) the release of microplastics that pollute air, water, and soil; (ii) the release of toxic substances through leaching; (iii) emissions of toxic gases that affect air quality; and (iv) the release of tire particles that affect the health of aquatic ecosystems (Mayer et al., 2024).

Despite the lack of alternatives to manage rubber waste in an environmentally sustainable way, biodegradation or biotransformation has emerged as a viable solution. One promising avenue is the enzymatic degradation of rubber using oxidative enzymes (Soares and Steinbüchel, 2022). Currently, three key enzymes have been characterized for their ability to cleave the carbon double bonds present in poly(*cis*-1,4-isoprene) through an oxidative mechanism. Among the most studied rubber oxygenases is latex clearing protein (Lcp), first identified in *Streptomyces* sp. K30 (Lcp_K30_) (Rose and Steinbüchel, 2005). Subsequently, Lcps were identified in bacteria such as *Gordonia polyisoprenivorans* VH2 (Lcp_VH2_), *Rhodococcus rhodochrous* RPK1 (Lcp_Rr_), and other *Streptomyces* species (Ilcu et al., 2017; Basik et al., 2021). The other group of rubber oxygenases are the rubber oxygenases (RoxA and RoxB), both isolated from *Steroidobacter cummioxidans* 35Y. RoxA mainly produces 4,8-dimethyl-12-oxotrideca-4,8-dienal (ODTD) (Braaz et al., 2005) as a degradation product through an exo-type cleavage mechanism. RoxB and Lcp generate oligoisoprenoids with different chain lengths by an endo-type cleavage mechanism (Jendrossek and Birke, 2019). Studies such as Jendrossek and Birke (2019) and Braaz et al. (2005) reported poly(*cis*-1,4-isoprene) cleavage products ranging from C_20_ to ≈ C_65,_ and Andler et al. (2022) and Braaz et al. (2005) reported 17 degradation products that were oligoisoprenoids from C_20_ to ≈ C_100_.

Three Lcps have been biochemically characterized: Lcp_VH2_ (Linos et al., 2000), Lcp_K30_ (Rose et al., 2005), and Lcp_Rr_ (Watcharakul et al., 2012). In addition, the crystal structures of Lcp_K30_, including the open state (PDB ID: 5O1L) and the closed state (PDB ID: 5O1M), have been reported (Ilcu et al., 2017). Comparing the open and closed structure of Lcp_K30_, a remarkable flexibility is observed in the protein at its active site. This flexibility suggests that residues Lys167 and Thr168 might undergo a conformational change from the closed to the open state upon substrate binding, which facilitates access to the distal axial position of the heme group of the enzyme, allowing substrate binding (Ilcu et al., 2017).

Quantum mechanical/molecular mechanics analysis indicates that the addition of dioxygen to the heme at the C=C double bond initiates the cleavage of the substrate via a dioxetane intermediate, a mechanism shared by heme dioxygenases such as indoleamine and tryptophan 2,3-dioxygenases (Ilcu et al., 2017). For Lcp_K30_, two possible routes for poly(*cis*-1,4-isoprene) scission have been proposed. In both, the abstraction of a proton with the participation of Glu148 as a catalytic base is crucial. In Route I, the distal oxygen atom of the dioxygen is added to the C=C double bond, forming an epoxide intermediate prior to the scission of the O-O bond. In Route II, the oxygen atom proximal to the iron attacks the C=C double bond, forming a dioxetane intermediate (Ilcu et al., 2017; Zhang and Liu, 2020). These mechanisms help us to understand how Lcp_K30_ and other heme dioxygenases catalyze the specific cleavage of their substrates by incorporating dioxygen.

Despite advances in biochemical characterization and bioinformatics analyses that seek to explain the catalytic mechanism of Lcp, the enzymatic kinetics have not been studied to date from the perspective of bioprocess engineering and enzyme kinetics. Such an approach is necessary given that the degradation rates of rubber polymers are still relatively low, reaching conversion yields of about 32% (Andler et al., 2022). In view of the above, this study aimed to analyze the enzymatic kinetics of Lcp_K30_ when using poly(*cis*-1,4-isoprene) as a substrate to determine specific parameters and understand the mechanism involved. For this purpose, oxygen consumption analyses were performed between the enzyme and the substrate under different conditions, linking the experimental data with theoretical mass balances. In addition, to understand the stability and conformational changes of the enzyme, molecular docking with different chain lengths was carried out to study the interaction of poly(*cis*-1,4-isoprene) with Lcp_K30_.

## 2 Materials and methods

### 2.1 Production of Lcp_K30_

For the synthesis of Lcp_K30_, the transformation of *Escherichia coli* BL21 (DE3) cells with the plasmid p4782.1::*lcp*_K30_ was performed. Cells were cultivated at a bioreactor scale (Minifors 2, Infors), using 4 L of Terrific Broth (TB) at 22°C, 0.5 vvm, and 500 rpm for 30 h. One liter of TB was prepared as follows: 24 g of yeast extract, 12 g of tryptone, 15.6 mL of 85% (v/v) of glycerol, 12.5 g of K_2_HPO_4_, and 2.3 g of KH_2_PO_4_. Cells were grown under kanamycin-resistant conditions with a concentration of 50 μg/μL and induction with 0.1% (w/v) of L-rhamnose. Cells were harvested with centrifugation at 3.011 × g for 20 min. The cell disruption was performed with an ultrasonic homogenizer (Hielscher, UP200st), using four cycles of 30 s at a power (P) of 20 W, pulse (C), and amplitude (A) of 50. The soluble fraction was obtained after centrifugation at 31.514 × g and 4°C for 1 h. Purification of Lcp_K30_ was performed using a 1 mL Strep-Tactin XT (IBA Lifesciences) gravity flow column. The column was washed with 2–3 CV (column bed volume) of NaOH freshly prepared, and then the column was equilibrated using 2–3 CV of buffer W (100 mM Tris-HCl at pH of 8.0, 150 mM NaCl, 1 mM EDTA). Elution of Lcp_K30_ was achieved after adding buffer BXT (Buffer W containing 6 mg biotin/mL). To assess the purity of the Lcp_K30_ enzyme, SDS-PAGE was performed using BIO-RAD gels. The gel was loaded with 8 μL of molecular weight marker and 5 μg of purified Lcp_K30_. Electrophoresis was conducted with 1X running buffer (25 mM Tris, 192 mM glycine, and 0.1% w/v SDS, pH 8.2) at 35 mA for approximately 1 h. After electrophoresis, the gel was stained with Coomassie Blue staining solution (0.25% w/v Coomassie Blue, 10% v/v acetic acid, and 45% v/v methanol) for 1 h. The gel was then decolorized with a decolorization buffer (10% v/v acetic acid and 10% v/v methanol in water) for approximately 24 h. Characterization of the absorption spectrum of Lcp_K30_ was conducted by UV-vis spectroscopy (UV-1900i, Shimadzu) through a scan ranging from 190 nm to 900 nm in order to detect the signal of the heme group.

### 2.2 Activity assay

The enzyme activity of Lcp_K30_ was determined by molecular oxygen consumption in a reaction containing a poly(*cis*-1,4-isoprene) latex emulsion as a substrate, provided by Carbios, France. For this, an oxygen electrode system (OXIG1 plus, Hansatech) with the software OxyTrace+ was used. The 100% oxygen saturation was calibrated with Tris-HCl 0.2 M at a pH of 7.2 and a constant agitation of 30 rpm, while the 0% concentration was calibrated using sodium sulfite. The assays were conducted at 30°C and 30 rpm for 1 h. The enzyme concentration was 200 μg/mL, and the following different NR concentrations (NR in mg/mL) were evaluated: 5.0, 10, 15, 20, 25, 30, 35, 40, 45, 50, and 55. As negative controls, two assays were performed: (i) in the absence of NR and (ii) NR at a concentration of 15 mg/mL after denaturation. Denaturation was performed at 95°C for 5 min in a dry bath (Thermo Fisher Scientific, D). The enzyme was added when a stable initial signal of 220 ± 20 nmol O_2_/mL was achieved. The volume of the latex emulsion (V_latex_) added to the assay was based on the NR content in the samples. For this, the following parameters were considered: latex density of 950 g/L, 25% (v/v), and a 60% theoretical content of poly(*cis*-1,4-isoprene) in latex. The latex was also diluted four times using Tris-HCl with a pH of 7.2.

### 2.3 Extraction and detection of oligoisoprenoids

To isolate the oligoisoprenoids from the reaction mixture, 1 mL of ethyl acetate (EA) was added to 1 mL of the reaction mixture and vigorously mixed for 1 min (IKA, Genius 3). Subsequently, it was centrifuged at 10.000 × g for 10 min (Hettich, Mikro 220R), and 600 µL of the upper phase containing the degradation products was recovered. The process was repeated by adding 600 µL of EA to the previously extracted sample, and a second extraction process was performed. A total of 1.2 mL containing the oligoisoprenoids was recovered, and the EA was then evaporated. Subsequently, the samples were analyzed using high-performance liquid chromatography (HPLC), following the methodology by Ilcu et al. (2017) and Braaz et al. (2005), at 23°C, with a RP-8 column model Kromasil 100-5-C8 (YL9100 Plus HPLC).

### 2.4 Determination of enzyme kinetic parameters

Initially, the results for a 1-h reaction period were plotted. Subsequently, the data from the first 200 s were selected and graphed separately to emphasize the initial phase of the reaction. Measurements that exhibited a clear linear relationship between molecular oxygen consumption and time were identified and utilized for further analysis. A linear regression was performed on these selected data to obtain the slope for each substrate concentration, representing the initial reaction velocity (V_0_). The Lineweaver–Burk equation was then employed to calculate the Michaelis–Menten constant (K_m_) and the maximum reaction velocity (V_max_).

### 2.5 Effect of oligoisoprenoids on the conversion process

Three assays were performed in a multichannel bioreactor (BioSan RTS-8) to evaluate the enzymatic activity of Lcp_K30_ in the presence of NR and the effect of degradation products or oligoisoprenoids, measuring on-line dissolved oxygen and pH profiles. In assay 1, the reaction was made up of 40 mg/mL NR 25% (v/v) and 200 μg/mL Lcp_K30_, adjusting the final volume to 10 mL with Tris-HCl pH 7.2. The solution was incubated at 150 rpm for 24 h in a 50 mL tube containing the dissolved oxygen and pH sensors. Upon completion, liquid–liquid extraction was performed, as mentioned in Section 2.3. For assay 2, 800 µL of the product of assay 1 was transferred to a new 50 mL reaction tube containing 40 mg/mL of NR 25% (v/v) and 200 μg/mL of Lcp_K30_, filling up to a final volume of 10 mL with Tris-HCl pH 7.2. Incubation was performed at 150 rpm for 24 h. Subsequently, a liquid–liquid extraction of this sample was carried out. For assay 3, the liquid–liquid extraction from assay 1 was used. This fraction was incorporated into a new reaction tube with a solution of 40 mg/mL NR 25% (v/v) and 200 μg/mL Lcp_K30_, adjusting the final volume to 10 mL with Tris-HCl pH 7.2. The mixture was incubated at 150 rpm for 24 h, followed by liquid–liquid extraction.

### 2.6 Mathematical modeling of enzyme kinetics

The enzymatic activity of Lcp_K30_ during the conversion of poly(*cis*-1,4-isoprene) was evaluated using the Michaelis–Menten model and the Hill model to describe the relationship between enzymatic reaction rate and substrate concentration. These models are given respectively by the following:

$$v=\frac{V_{\max}\left[S\right]}{K_{m}+\left[S\right]}$$ (1)

$$v=\frac{V_{\max}{\left[S\right]}^{n}}{K_{m}^{n}+{\left[S\right]}^{n}}$$ (2)

where v is the reaction rate, [S] is the substrate concentration, V_max_ is the maximum reaction rate, K_m_ is the Michaelis–Menten constant, and n is the Hill coefficient, which indicates the degree of cooperativity (Goutelle et al., 2008; Baldera-Moreno et al., 2022). The parameter values were estimated by a nonlinear fit to the experimental data.

### 2.7 Theoretical calculations using the Fukui function

The Fukui function (Parr and Yang, 1984) is a local reactivity descriptor that measures the change of the electron density induced by the change of the number of electrons with a fixed geometry and potential, which can be used to identify the reactive sites of a molecule. The Fukui function of short-chain polyisoprene and oligoisoprenoid was computed under the finite difference approximation, where the Fukui index associated with free radical attacks $f^{0}$ can be written as follows:

$$f^{0}\left(r\right)≅\frac{1}{2}\left[\rho_{N+1}\left(r\right)-\rho_{N-1}\left(r\right)\right]$$ (3)

where r stands for the three-dimensional coordinates, N is the total number of electrons of the neutral system, and $\rho {\left(r\right)}_{N+1}$ and $\rho {\left(r\right)}_{N-1}$ are the electron densities of the anion ( N+1 ) and cation ( N−1 ) systems, respectively, in the same geometry as the neutral species. The condensed Fukui function $f_{c}$ can defined based on finite differences of the atomic charges (Yang and Mortier, 1986; Ayers et al., 2002), which allow partitioning of the electron density $\rho \left(r\right)$ between the atoms of the molecule. Like Equation 1, the condensed Fukui index for free radical attacks of the *i*th atom $f_{i}^{0}$ is defined as follows:

$$f_{i}^{0}≅\frac{1}{2}\left[q_{i}\left(N+1\right)-q_{i}\left(N-1\right)\right]$$ (4)

where $q_{i}\left(N+1\right)$ and $q_{i}\left(N-1\right)$ are the partial charges of the i -th atom in the anion ( N+1 ) and cation ( N−1 ) species, respectively.

The tridimensional structures of the molecules were sketched in Schrödinger’s Maestro visualization software. Up to 64 conformers were generated with the Conformator program (Friedrich et al., 2019) using the “Best” mode, and the lowest energy conformation was further optimized by the Density Functional Theory (DFT) method. Single-point calculations of the anion and cation species were performed with the optimized structure. DFT calculations were carried out at the ωB97X-D4 (Najibi and Goerigk, 2020; Chai and Head-Gordon, 2008)/def2-TZVP (Weigend and Ahlrichs, 2005) level of theory in the gas phase using the ORCA v6.0 software (Neese et al., 2020). The default RI-J approximation for Coulomb integrals and COSX numerical integration for HF exchange (RIJCOSX) (Helmich-Paris et al., 2021) were enabled to speed up the calculations. Atomic partial charges were calculated with the Hirshfeld method (Hirshfeld, 1977) because it has been shown to be the most adequate for computing the condensed Fukui function (Roy et al., 1999; Roy et al., 2000; De Proft et al., 2002).

### 2.8 Calculations

The following parameters were measured: volumetric activity, specific activity, volumetric productivity, and specific production.

$$\text{Volumetric}\;\text{activity}=\frac{U}{V_{\text{Lcp}}}$$ (5)

$$\text{Specific}\;\text{activity}=\frac{U}{M_{\text{Lcp}}}$$ (6)

$$\text{Lineweaver}–\text{Burk}=\frac{1}{V_{0}}=\frac{K_{m}}{V_{\max}\times \left[S\right]}+\frac{1}{V_{\max}}$$ (7)

$$\text{Volumetric}\;\text{productivity}\;\left(Q_{\text{Lcp}}\right)=\frac{M_{\text{Lcp}}}{V\times t}$$ (8)

$$\text{Specific}\;\text{production}\;\left(q_{\text{Lcp}}\right)=\frac{1}{X}\frac{M_{\text{Lcp}}}{t}$$ (9)

where U represents the unit of enzymatic activity (μmol min^−1^), V_Lcp_ denotes the volume of the sample (mL) containing the enzyme, M_Lcp_ indicates the mass of added Lcp_K30_ (mg), V is the cultivation volume (L), t is the cultivation time (h), and X is the cell concentration (g L^−1^).

## 3 Results and discussion

### 3.1 Production of Lcp_K30_ at bioreactor scale

**FIGURE 1:**
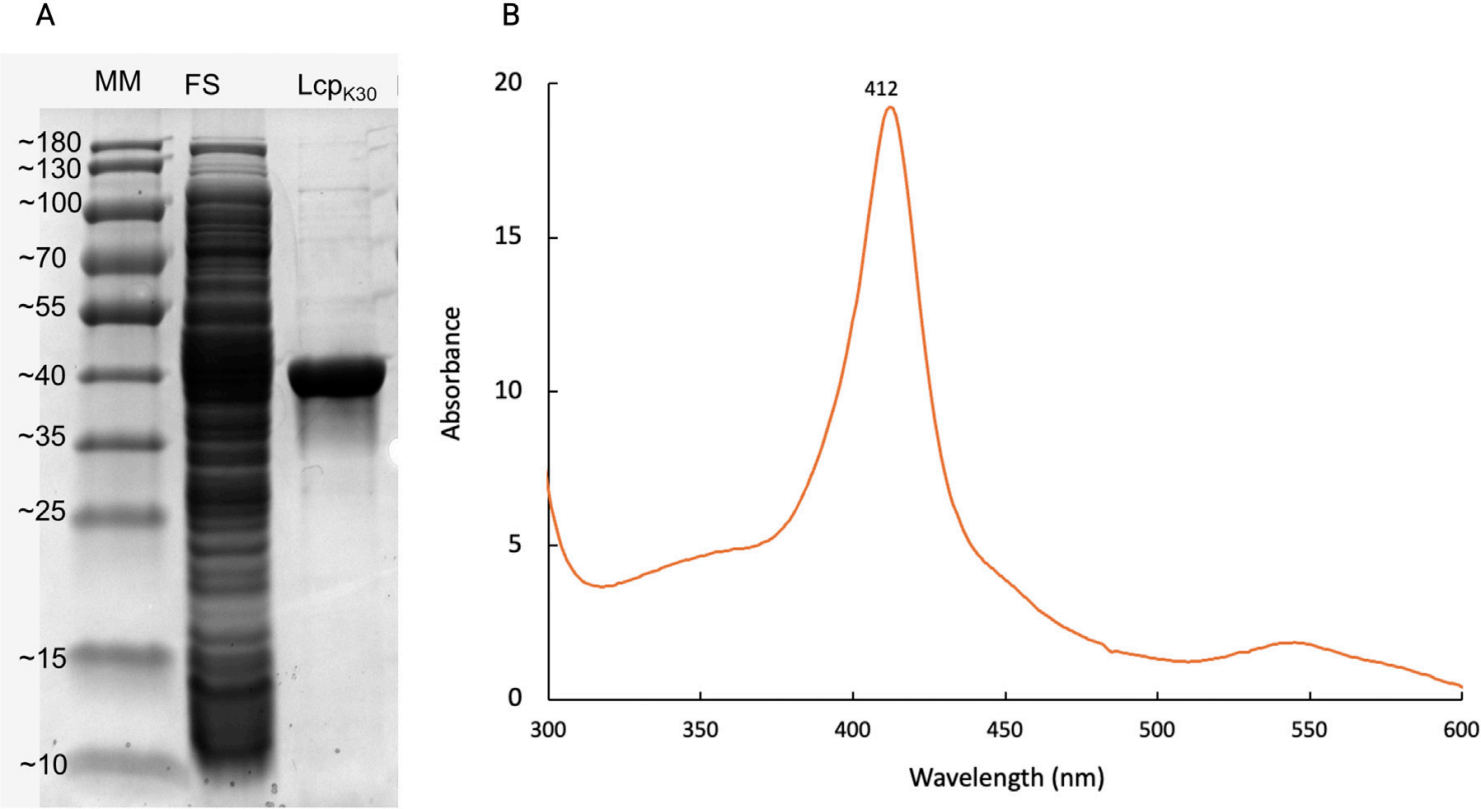
Purity analysis of Lcp_K30_ and detection of its heme cofactor. **(A)** SDS-PAGE under denaturing conditions showing the molecular weight marker (180 kDa), the soluble protein fraction, and 5 µg of purified Lcp_K30_ obtained from cultures grown in TB medium. A predominant band at approximately 40 kDa is observed, consistent with the theoretical molecular weight of Lcp_K30_. **(B)** UV-Visible absorption spectrum of the purified protein, showing a distinct peak at 412 nm, characteristic of a heme cofactor, supporting its presence in the active form of Lcp_K30_.

The synthesis of Lcp_K30_ using *E. coli* BL21 (DE3) + p4782.1::*lcp*_*K30*_, was carried out in 4-L bioreactors. For the first time, a culture for the production of Lcp_K30_ using TB medium was performed with a heterologous expression mechanism using L-rhamnose. At the end of the culture, a cell biomass of 4.58 ± 0.01 g/L was obtained, reaching a volumetric productivity of 0.05 (mg/Lh) (Equation 6) and a specific productivity of 0.04 (mg/gh) (Equation 7). After purifying the protein with Strep-Tactin XT, 6.1 ± 0.4 (mg/L) of Lcp_K30_ was obtained, and ≈24.2 mg of the enzyme was obtained in the total culture. These results were similar to previous studies, where values of 5.4 mg Lcp_K30_ per liter in a shake flask (Birke et al., 2015) and 6.2 mg Lcp_Rr_ per liter in a stirred tank bioreactor have been reported, also using rhamnose-inducible expression systems (Andler et al., 2022). In terms of biomass yield in the product, approximately 1.33 mg/g was obtained. To analyze the purity of Lcp_K30_, an SDS-PAGE was performed, where bands of approximately 42 kDa (Figure 1A), characteristic of Lcp_K30_, were observed. Additionally, a UV-vis spectroscopic analysis was performed, which revealed an intense absorption band at 412 nm (Figure 1B), which coincides with that reported by Birke et al. (Braaz et al., 2005). Optimizing cultivation conditions, particularly agitation rate and cultivation time, could significantly improve Lcp_K30_ production yields. Proper adjustment of agitation rate would enhance oxygenation and medium mixing, promoting more efficient cell growth. Likewise, modifying cultivation time could help maximize protein expression without compromising cell viability, leading to higher productivity and reducing potential negative effects on the cells.

### 3.2 Enzyme kinetics of Lcp_K30_

#### 3.2.1 Substrate degradation kinetics

**FIGURE 2:**
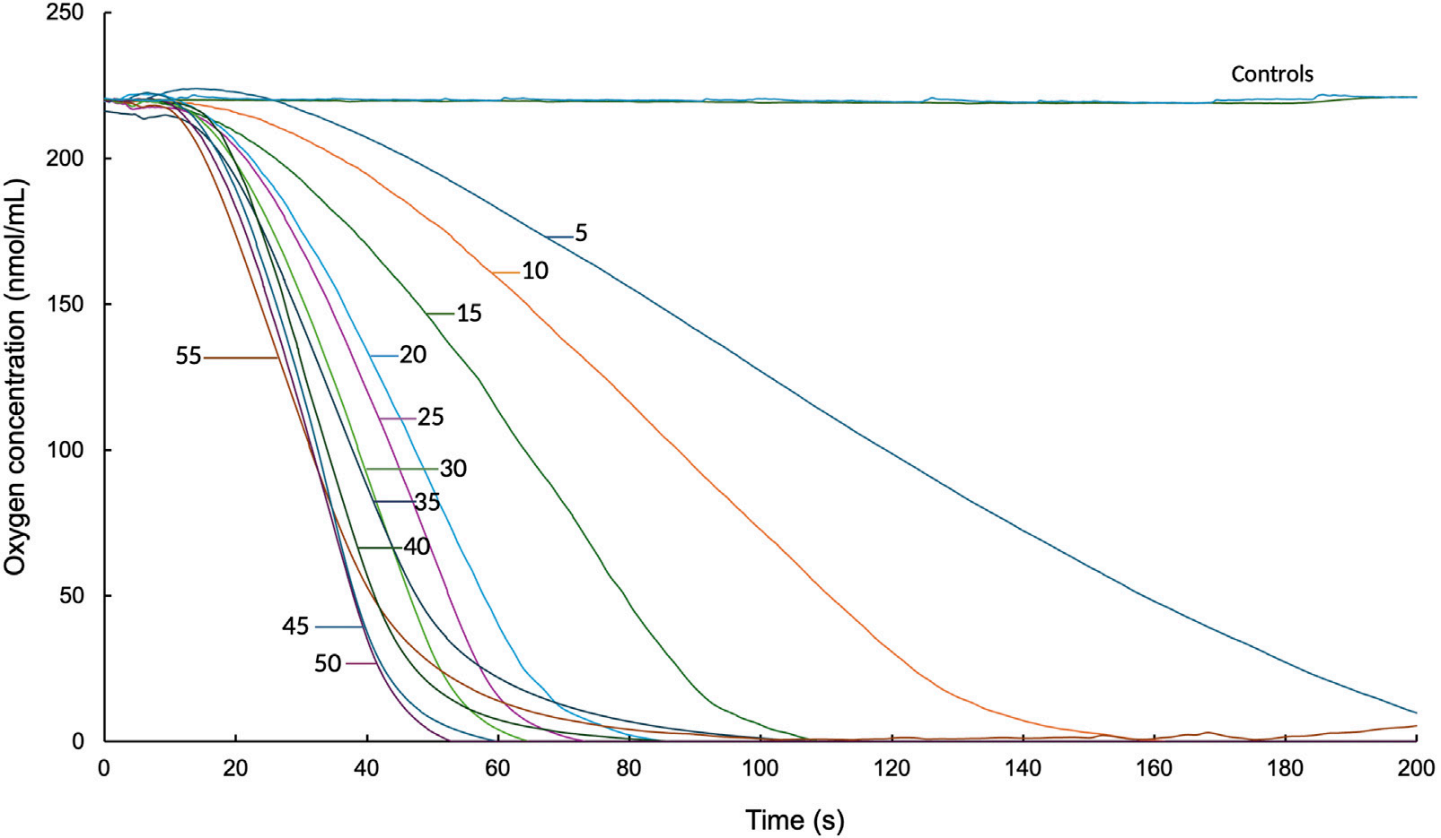
Oxygen consumption assays by Lcp_K30_ (200 μg/mL) in the presence of different poly(*cis*-1,4-isoprene) concentrations (5–55 g/L) over a 200-s reaction time.

The results indicated a positive correlation between initial velocity and substrate concentration. As the substrate concentration increased, an increase in the initial reaction rate was observed. Control 1, which consisted of 0.2 M Tris-HCl and latex milk, and control 2, which included heat-inactivated protein together with 0.2 M Tris-HCl and latex milk, showed that there was no oxygen consumption under these conditions, as indicated by the literature (Hiessl et al., 2014). In the analysis of the data obtained from Figure 2, the initial velocity was calculated for each concentration of poly(*cis*-1,4-isoprene), reaching a maximum initial velocity of 7.05 nmol O_2_/min with a substrate concentration of 55 mg/mL.

Figure 2 presents the graph of the initial reaction rate. It shows that a substrate concentration of 55 mg/mL results in a saturation point, with an initial rate (V_0_) of 6.84 nmol O_2_/min. Notably, 55 mg/mL is equivalent to 5 μg Lcp_K30_ per mg NR.

Equation 7 was used to plot the reciprocals of the obtained data where the relationship between poly(*cis*-1,4-isoprene) concentration and reaction V_0_ was evaluated by an oxygen consumption assay (Figure 2). The data obtained suggest that the initial reaction rate increases with substrate concentration until a saturation threshold is reached. At a concentration of 55 mg/mL poly(*cis*-1,4-isoprene), a maximum velocity (V_max_) of 59.2 nmol O_2_/min was observed, indicating that the enzyme reached its maximal catalytic capacity. This behavior is consistent with enzyme saturation, a condition in which all active sites are occupied by substrate molecules, such that further increases in substrate concentration do not enhance the reaction rate. Therefore, the observed plateau is attributed to enzyme saturation rather than substrate inhibition. This interpretation is supported by the Michaelis–Menten constant (K_m_) determined for Lcp_K30_, which was 308.3 mg/mL. Since the maximum velocity was reached at 55 mg/mL, a concentration substantially lower than the K_m_, no evidence of substrate inhibition was observed under the conditions evaluated.

**FIGURE 3:**
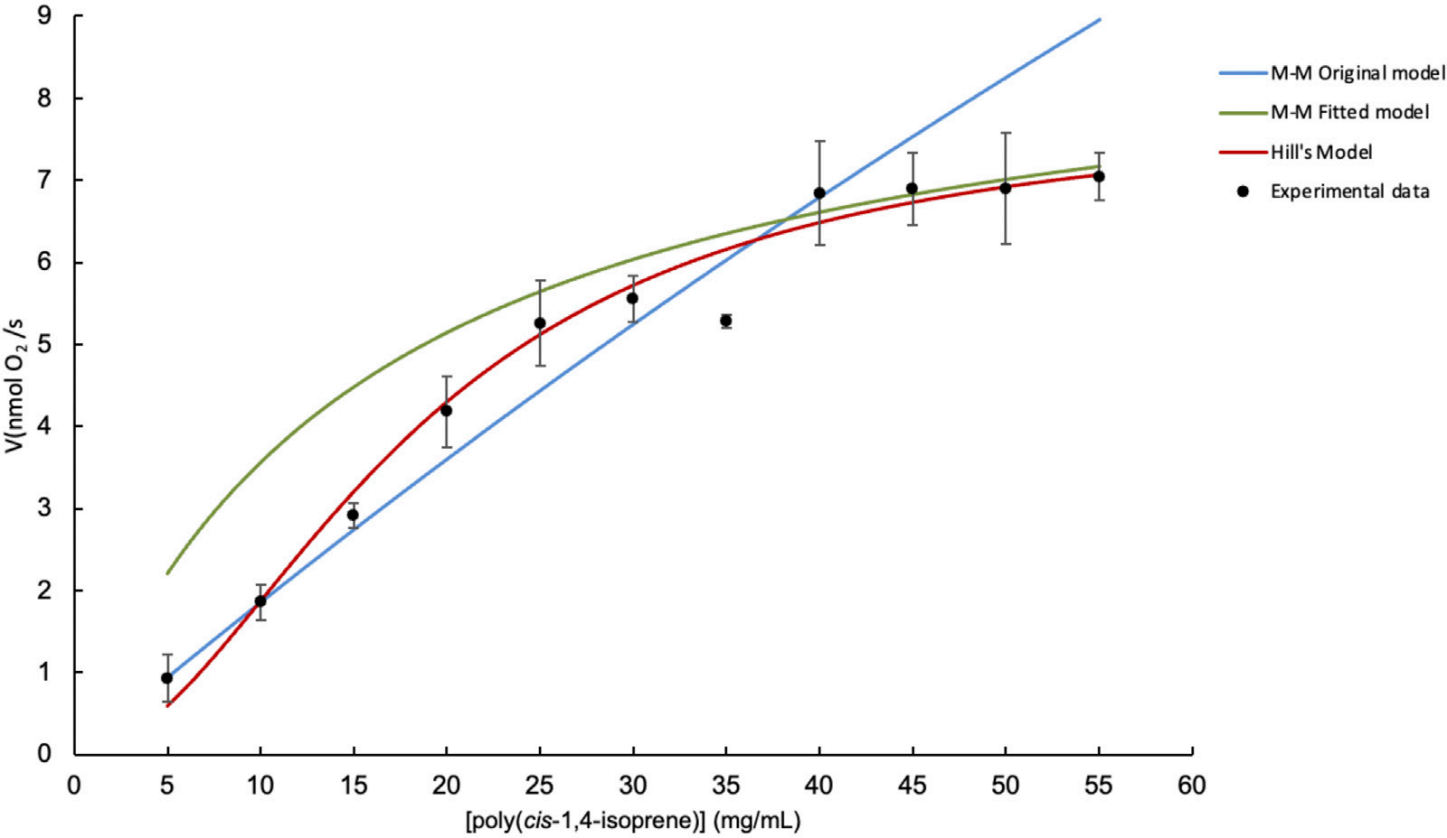
Comparison of kinetic models for the evaluation of Lcp_K30_ initial reaction rate versus poly(*cis*-1,4-isoprene) concentration. Michaelis–Menten model (blue), adjusted Michaelis–Menten model (green) and Hill model (red).

Figure 3 shows a comparison between three kinetic models fitted to experimental data evaluating the initial reaction rate (V_0_) of Lcp_K30_ versus poly(*cis*-1,4-isoprene) substrate concentration. The models represented are the original Michaelis–Menten model (blue curve), the adjusted Michaelis–Menten model (green curve), and the Hill model (red curve) (Equation 2).

The original Michaelis–Menten model, which has a V_max_ = 59.01 (nmol O_2_/s) and K_m_ = 307.43 (mg/mL) with an *R* ^2^ = 0.8374, does not adequately describe the reaction rate of the Lcp_K30_ enzyme. At low and medium substrate concentrations (5–30 mg/mL), the model predicts lower reaction rates than actually observed, which is because the high K_m_ indicates that the enzyme does not have a good affinity for the substrate. At higher concentrations (30–55 mg/mL), the model predicts that the reaction rate continues to increase, reaching a value higher than what is observed experimentally, indicating that the model does not capture the saturation of the enzyme. Therefore, this model does not fit the experimental data well over the entire range of substrate concentrations.

The Michaelis–Menten model with fitted parameters (V_max_ = 14.49, K_m_ = 52.34) significantly improves the fit compared to the original model, with an *R* ^2^ = 0.9641. Although it better represents the experimental data at most concentrations, at lower concentrations, it still shows some deviation.

Hill’s model (V_max_ = 8.6562, K_m_ = 21.2454, n = 1.6458) shows the best fit to the experimental data with an *R* ^2^ = 0.9784. As can be seen, the red curve closely follows the trend of the experimental data over the entire range of substrate concentrations, capturing both the initial phase of rate increase and saturation at higher concentrations. This suggests that positive cooperativity between substrate binding sites could be relevant in this enzyme system.

The observed cooperativity, as described by the Hill model, suggests that Lcp_K30_ may exhibit positive cooperativity in its substrate binding. This behavior could be explained by the formation of oligomeric enzyme complexes, where the binding of one substrate molecule to an active site may enhance the affinity for additional substrate molecules at other active sites. Furthermore, this cooperativity could arise from allosteric regulation, where the enzyme undergoes conformational changes upon substrate binding, increasing its affinity for subsequent substrates (Leskovac, 2003).

Therefore, Hill’s model is the best fit for the experimental data, as indicated by its high *R* ^2^ value and ability to closely follow the observed trend in reaction rates over the entire range of substrate concentrations.

#### 3.2.2 Product formation kinetics

**FIGURE 4:**
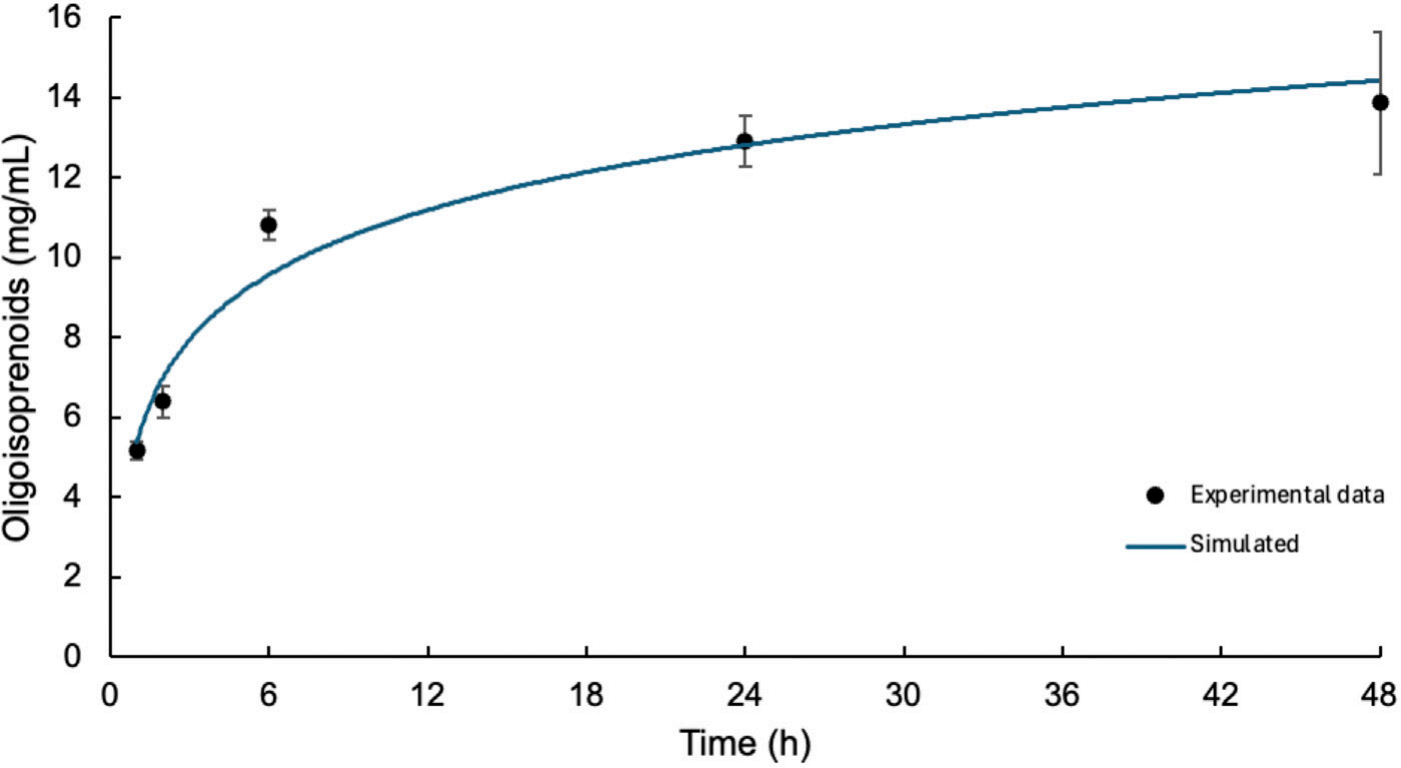
Formation of oligo(*cis*-1,4-isoprene) as a function of reaction time upon incubation at a concentration of 5 μg Lcp_K30_/mg NR. The blue line shows the logarithmic function that fits the experimental data.

The saturation point of Lcp_K30_ was determined, and an enzyme kinetics assay was performed to measure oxygen consumption at different time intervals. The cleavage products generated in this assay were extracted by liquid–liquid extraction with EA. After extraction and complete evaporation of the solvent, oligo(*cis*-1,4-isoprene) molecules were quantified by HPLC, with peaks corresponding to the various degradation products generated, similar to those previously reported (Andler et al., 2022; Birke et al., 2017). Figure 4 shows the formation of oligo(*cis*-1,4-isoprene) at different reaction intervals, showing a stabilization in oligomer production after 24 h. The experimental data were fitted to a logarithmic function, represented by the blue line, with the equation y = 2.3389 ln(x) + 5.3738. The model demonstrates an excellent fit to the experimental data, as indicated by the coefficient of determination (*R* ^2^ = 0.9617).

As expected, quantification of the resulting oligo(*cis*-1,4-isoprene) molecules showed an increase in the concentration for longer incubation periods with a maximum of 13.48 ± 1.78 mg in the 48-h enzyme activity assay. The concentrations observed at other intervals were as follows: 6.39 ± 0.40 mg at 2 h, 10.82 ± 0.38 mg at 6 h, and 12.91 ± 0.64 mg at 24 h. This maximum value significantly exceeds those reported in previous studies, where degradation products of approximately 4 mg were recorded (Andler et al., 2022). It is important to note that, despite extending the incubation times, no significant change was observed in the degradation pattern. In other words, longer incubation times did not result in the production of smaller oligo(*cis*-1,4-isoprene) molecules. This finding contrasts with what would be expected if the enzymatic action were to continue breaking down the generated products into smaller fragments.

#### 3.2.3 Oligoisoprenoids as a substrate for Lcp_K30_

**FIGURE 5:**
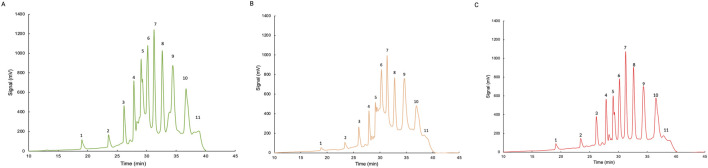
HPLC analysis of poly(*cis*-1,4-isoprene) cleavage products after enzymatic reaction with Lcp_K30_. **(A)** E.1 Assay containing 40 mg/mL NR, Tris-HCl, 200 μg/mL of Lcp_K30_. **(B)** E.2 Assay containing 800 µL of E.1 after incubation, 40 mg/mL NR, Tris-HCl, 200 μg/mL Lcp_K30_. **(C)** E.3 Assay containing dried degradation products of E.2, 40 mg/mL NR, Tris-HCl, 200 μg/mL Lcp_K30_.

The effect of oligo(*cis*-1,4-isoprene) molecules on the cleavage reaction of poly(*cis*-1,4-isoprene) by Lcp_K30_ was evaluated in three separate 24-h enzymatic reactions under different conditions. Assay 1 involved the reaction of 40 mg/mL of NR without oligoisoprenoids, while assays 2 and 3 contained oligoisoprenoids in liquid or dried form, respectively (details provided in Section 2.5). The amount of oligoisoprenoids after liquid–liquid extraction was quantified by weight, yielding the following values: assay E.1, 40.7 ± 0.16 mg; assay E.2, 36.9 ± 0.04 mg; and assay E.3, 36.1 ± 0.20 mg. HPLC analysis revealed a similar peak pattern, consistent with the data shown in Figure 5, with the HPLC chromatogram indicating the separation of oligo(*cis*-1,4-isoprene) molecules from C_20_ to C_65_. The 10 identified peaks should correspond to oligos from C_20_ (*n* = 3 units) to C_65_ (*n* = 12 units). Relative quantification based on these peaks indicated that assay 2 exhibited the highest value, accounting for 43.34% of the total area under the curve. This result may be explained by the additional NR present in this assay, likely derived from residual oligomers in the sample used for assay 1. In comparison, assay 1 accounted for 32.14%, while assay 3 showed the lowest percentage at 24.52%.

Additionally, it is important to consider the potential for product-related effects in these reactions. While no clear evidence of such effects was observed, the higher concentration of oligoisoprenoids in assay 2, compared to assay 3, may have influenced enzyme efficiency. However, since assay 2 contained more substrate due to the presence of residual latex, the observed differences in peak areas from the HPLC analysis might reflect a higher concentration of oligoisoprenoids in this sample rather than a direct product-related effect. Therefore, although the data show some variations in peak distribution, no definitive conclusions can be drawn regarding potential product-related effects.

The data obtained from gel permeation chromatography (GPC) analysis of the lyophilized samples from reactions E.1, E.2, and E.3 show that the conversion percentages of poly(*cis*-1,4-isoprene) to oligoisoprenoids are higher than those achieved by liquid–liquid extraction with EA, reaching values of ∼82%, ∼83%, and ∼84%, respectively, after 24 h of reaction time. These degradation percentages are reported for the first time in the context of these enzymatic reactions.

**FIGURE 6:**
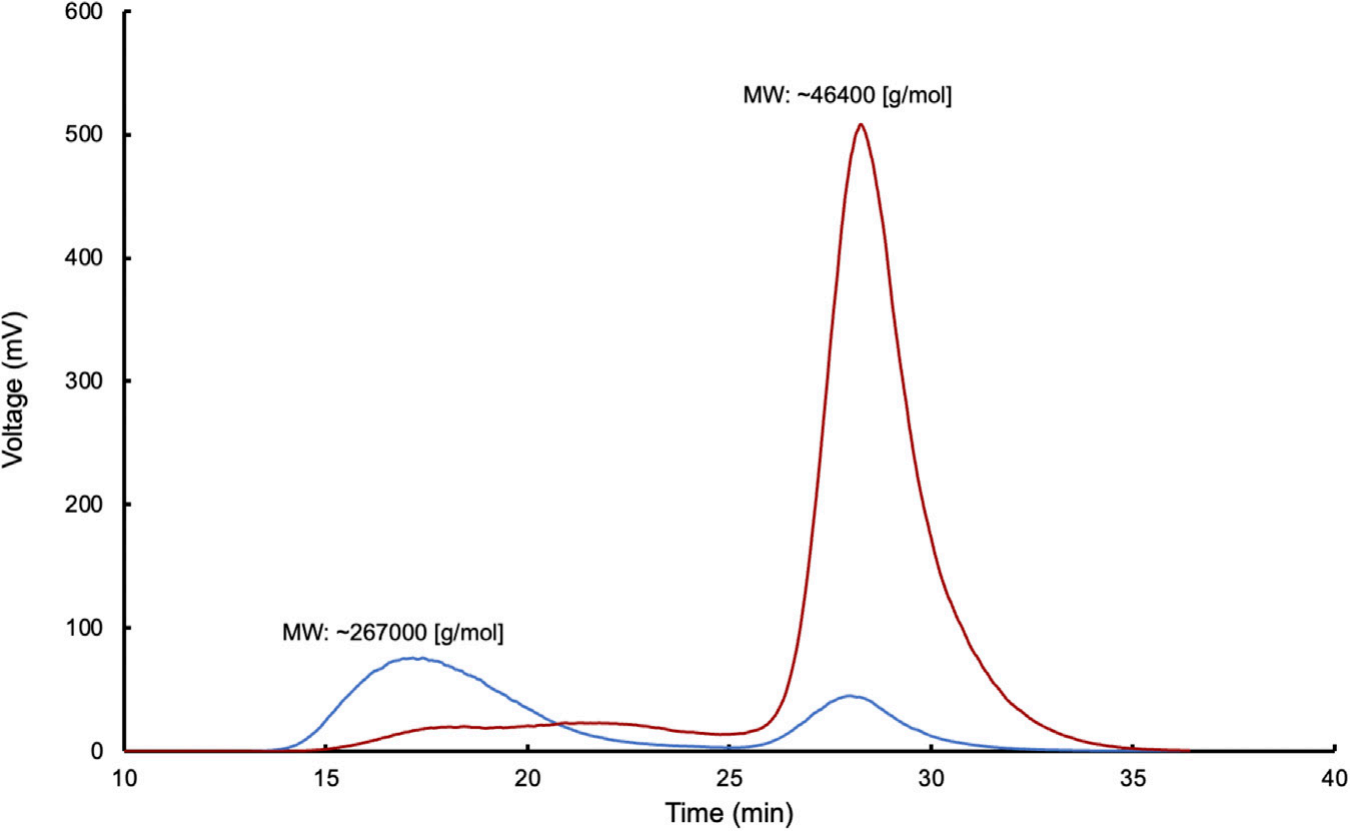
Gel permeation chromatography (GPC) analysis of cleavage products from reactions control (blue) and E.1 of poly(*cis*-1,4-isoprene) (red) after enzymatic reaction with Lcp_K30_ in a multichannel bioreactor.

Regarding the molecular weight (MW) of the samples, the observed values were as follows: for NR, the MW was ∼270,000 [g/mol]; for E.1, the MW was ∼46,400 [g/mol]; for E.2, the MW was ∼45,000 [g/mol]; and for E.3, the MW was ∼42,000 [g/mol]. Figure 6 presents the corresponding GPC chromatograms for these enzymatic reactions with Lcp_K30_, showing the GPC of the control (NR) and assay 1.

The discrepancy observed between the liquid-liquid extraction results and the GPC data can be attributed to differences in the efficiency of the methods employed. A key factor that may explain this discrepancy is the efficiency of the extraction process. Specifically, EA, a solvent of moderate polarity, may not be sufficiently effective in solubilizing all degradation products, especially those with diverse MWs and polarities (Andler et al., 2018). This may lead to an underestimation of the actual amount of products present in the samples.

In contrast, the sample freezing and subsequent lyophilization process allowed for a more complete recovery of the degradation products by avoiding the limitations associated with solvent-product affinity. Lyophilization, which is independent of solvent polarity, allowed for the recovery of a broader range of degradation products, regardless of their molecular weight or polarity. This process provided a more accurate characterization of the spectrum of oligoisoprenoids generated in these enzymatic reactions, ensuring a more reliable estimation of conversion rates. This approach minimized the risk of underestimating the total degradation products, thus capturing the full extent of enzymatic conversion.

**FIGURE 7:**
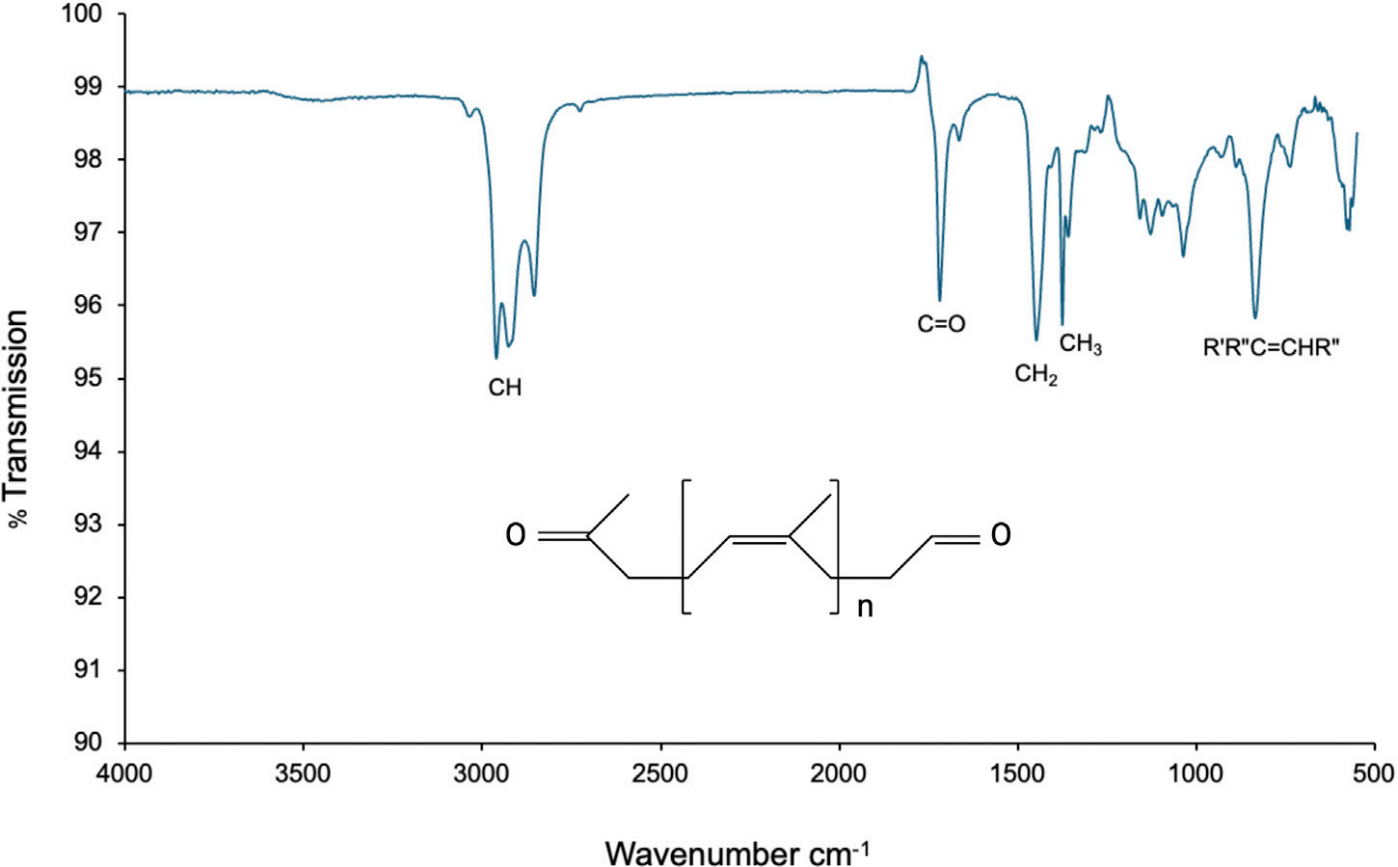
FTIR spectrum of the degradation products obtained after enzymatic conversion of latex with active Lcp_K30_. The spectrum shows bands corresponding to CH, CH_2_, CH_3_, and R′R″C=CHR″ groups, as well as a carbonyl (C=O) band at 1718.69 cm^−1^, indicating the formation of oxygenated cleavage products.

Additionally, the degradation products were analyzed by Fourier transform infrared spectroscopy (FTIR), which revealed the presence of CH, CH_2_, CH_3_, and R′R″C=CHR″ groups, confirming the formation of oxygenated compounds. A band at 1718.69 cm^−1^, corresponding to carbonyl (C=O) groups, also indicated the generation of cleavage products during the degradation process (Figure 7). This spectral profile is consistent with that previously reported by Hiessl et al. (2014) for natural rubber degradation products mediated by Lcps.

#### 3.2.4 Challenges in substrate complexity and enzymatic processing

One of the main challenges for the enzymatic transformation of rubber materials at an industrial scale is the use of complex substrates. While this study employed natural latex as a simplified model substrate for kinetic analyses, industrial applications must address the structural complexity of vulcanized rubber, which is highly cross-linked and contains a variety of chemical additives (Andler, 2020). These compounds, which are essential for tuning the mechanical properties and durability of rubber, can negatively affect enzymatic activity. Among the most critical additives such as the antioxidant N-(1,3-dimethylbutyl)-N′-phenyl-p-phenylenediamine (6-PPD) (Tian et al., 2022), vulcanization accelerators such as tetramethylthiuram disulfide (TMTD) and tetramethylthiuram monosulfide (TMTM), all of which have been shown to inhibit the growth of rubber-degrading microorganisms in small amounts (Altenhoff et al., 2019). The presence of such additives represents a major obstacle for the application of enzymes like Lcp_K30_ in complex rubber matrices. Consequently, the development of pretreatment protocols becomes essential to improve the accessibility and compatibility of these substrates for enzymatic processing.

Enzymatic stability is crucial for scaling up enzymatic transformation processes. In this study, Lcp_K30_ maintains its catalytic activity as long as the substrate is present, with prolonged activity observed at higher latex concentrations. In the experiments conducted with multichannel reactors, dissolved oxygen levels remained close to zero for approximately 7 h, confirming continuous oxygen consumption by the enzyme. This sustained oxygen consumption suggests that Lcp_K30_ efficiently utilizes oxygen for the conversion process over extended periods. These results highlight the potential of Lcp_K30_ in large-scale bioconversion processes, where enzyme stability and sustained activity are critical for efficient substrate conversion. However more studies need to be conducted to stablish the enzyme thermostability at different temperatures, replicating potential temperatures at an industrial scale.

For studies focused on biochemical characterization, enzyme purity is important, therefore the cost of purification is high. However, when scaling up the process, the purification can be replaced with salt precipitation with ammonium sulfate, reducing the cost (Andler and Steinbüchel, 2017). Also, the use of L-rhamnose as and inductor is expensive, nevertheless auto induction media (AIM) can produce higher cell densities and minimizing the cost.

### 3.3 Reactivity of the degradation products

**FIGURE 8:**
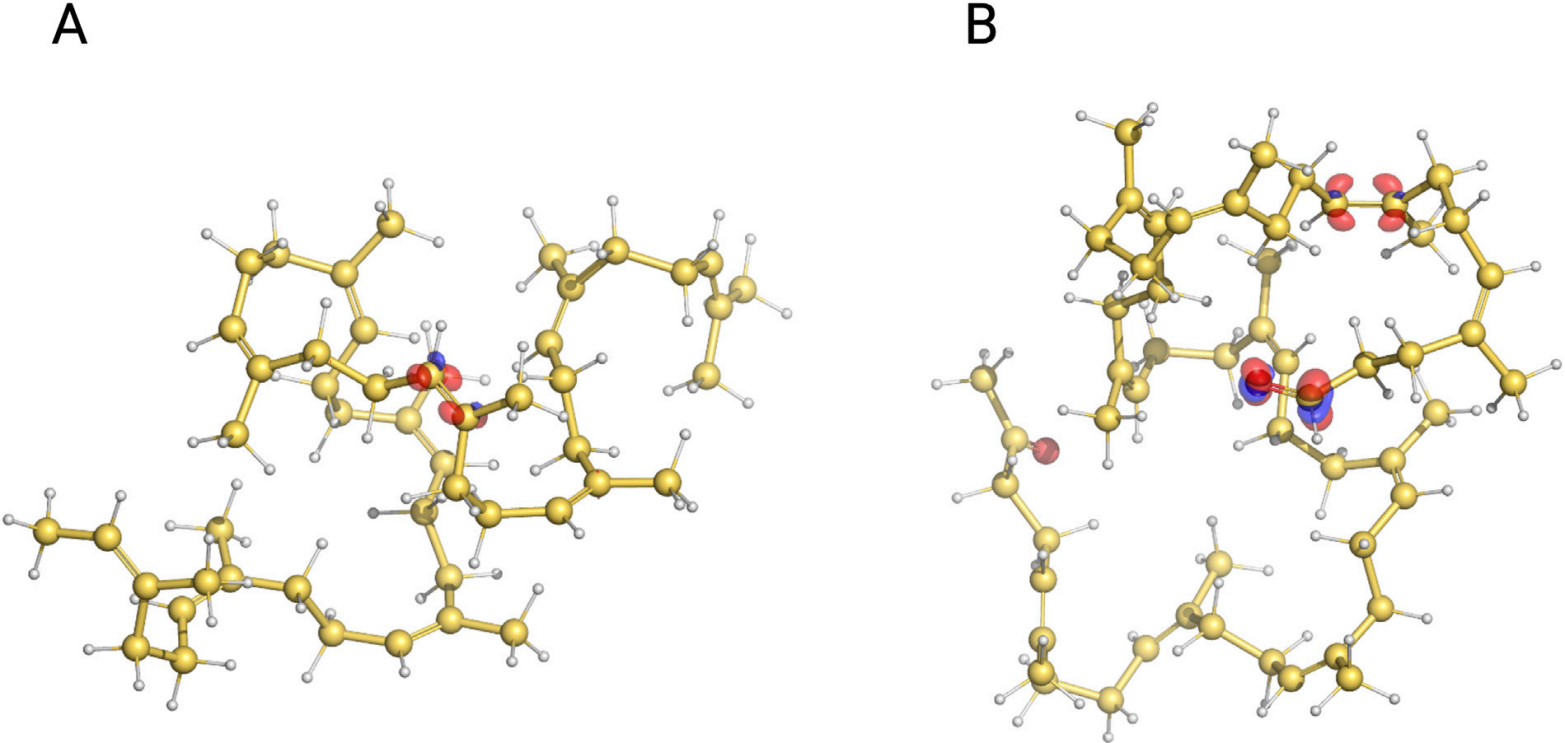
Isosurfaces of the Fukui function associated with radical attack $f^{0}$ projected onto the molecular structure of short-chain polyisoprene **(A)** and oligoisoprenoid **(B)**. Positive and negative values are shown in red and blue, respectively. Isosurfaces are drawn at the value of 0.01. Carbon atoms are shown in yellow, hydrogen in white, and oxygen in red.

Lcp cleavages NR by oxidizing double bonds at random along the polyisoprene chain, producing oligoisoprenoids of different lengths (C_20_ and larger) with terminal keto or aldehyde groups (Andler et al., 2018). We found that the main cleavage products are C_55_–C_65_, regardless of the substrate and enzyme concentrations and incubation times (Figure 8). One possible interpretation of this outcome is that oligoisoprenoids cannot undergo further cleavage; shorter (C_20_–C_25_) chains would be the most abundant product. Such inactivity could be related to the diminished ability or even inability of these molecules to bind to the enzyme due to the presence of the terminal groups, although it is difficult to probe due to the lack of structural information and the limitations of molecular modeling to simulate the binding of large molecules. Alternatively, the chemical properties of small molecules can be accurately computed by quantum chemistry calculations, which could be used to evaluate whether oligoisoprenoids are less susceptible to oxidization. Local reactivity descriptors such as the Fukui function (Parr and Yang, 1984) have been widely used to determine the reactivity and regioselectivity of reactant molecules of chemical reactions (Pucci and Angilella, 2022). The regions of a molecule with the largest values of the Fukui function are suitable for nucleophilic, electrophilic, or radical attacks. It has been suggested that the Lcp enzymatic reaction starts with a radical attack of the distal oxygen of the heme-bound dioxygen to the double bond of the substrate, followed by the formation of a dioxetane intermediate and eventual collapse into the final ketone and aldehyde (Zhang and Liu, 2020). The first step of the reaction is proposed to be the rate-limiting step. Therefore, we evaluated the Fukui function associated with a radical attack $f^{0}$ (Equation 3) in short-chain polyisoprene (c_55_) and oligoisoprenoid (C_55_) containing ten repeating isoprene units. We also tested shorter chains (five units) similar to Zhang and Lui’s work, and the results were quite similar (data not shown). The $f^{0}$ is projected onto the molecular structure of both molecules (Figure 8), and the atom-condensed Fukui function $f_{i}^{0}$ (Equation 4) of every atom is listed in the Supplementary Table S1. As may be expected, $f^{0}$ is primarily located in the carbon atoms of the double bond in the oligoisoprene molecule (red surfaces in Figure 8A), denoting that such a region is the most susceptible to a radical attack. Notably, one double bond (the seventh isoprene unit) seems to be the only reactive bond, where the associated carbon atoms (C2 and C3 in Supplementary Table S1) have $f_{i}^{0}$ values of 0.09 and 0.07, respectively, while the rest are close to zero. Likewise, the $f^{0}$ of the oligoisoprenoid molecule (Figure 8B) is located in one double bond (ninth isoprene unit) with a very similar $f_{i}^{0}$ values (0.08 and 0.07, respectively). However, $f^{0}$ is also observed at the terminal aldehyde group with $f_{i}^{0}$ values of the carbon (C7) and oxygen (OXT) atoms of the tenth isoprene unit equal to 0.13 and 0.11, respectively. This result suggests that both the double bond and aldehyde group are susceptible to a radical attack. Furthermore, the larger $f_{i}^{0}$ values of the aldehyde group imply that it may be favored over the double bond upon the reaction with the activated dioxygen molecule. The attack on the aldehyde group would prevent the formation of the dioxetane intermediate, rendering the enzyme unable to break the double bond. Consequently, it could be inferred that once oligoisoprenoids are formed, they will not be subject to enzymatic degradation or, at least, be degraded at a much lesser rate, which somewhat explains the production of chains of different lengths.

## 4 Conclusion

For the first time, the enzymatic kinetics of Lcp_K30_ for the degradation of NR have been characterized. The implementation of a multichannel bioreactor with precise control of temperature and aeration allowed for accurate measurement of oxygen consumption and subsequent quantification of substrate conversion, achieving conversion rates higher than those previously reported. Kinetic characterization through the Michaelis–Menten constant (K_m_) and the Hill model revealed that the enzyme becomes saturated at substrate concentrations close to 55 mg/mL, providing critical information on its catalytic capacity. Additionally, analysis using the Fukui function identified reactive sites in the oligoisoprenoids, suggesting that aldehyde terminal groups are less susceptible to enzymatic degradation. These findings open the possibility of developing an efficient bioprocess and a scaling strategy for the production and functionalization of oligoisoprenoids derived from NR degradation by Lcp_K30_, with the aim of generating new high-value-added polymeric materials.

## Data Availability

The raw data supporting the conclusion of this article will be made available by the authors, without undue reservation.
