# Supplementary material for: New insights on poly(cis-1,4-isoprene) rubber degradation through enzymatic kinetics and process improvement
Source: Front Bioeng Biotechnol. 2025 May 2;13:1593339. doi: 10.3389/fbioe.2025.1593339 (PMC12081455; doi:10.3389/fbioe.2025.1593339)
Supplement: Supplementary file 1 [file Table1.docx]

# **Supplementary material**

**Table S1**. Condensed Fukui indices for radical attacks $f_{i}^{0}$ based on Hirshfeld charges of short-chain polyisoprene (c_55_) and oligoisoprenoid (C_55_). Labels indicate atom names, and bold numbers (#) indicate isoprene units (10 in total). Bold values indicate atoms involved in the double bond, and italicized values indicate atoms belonging to the terminal keto and aldehyde groups. Values are colored from white (zero) to red (maximum value).

| **#** | **Atom** | **c55** | **C55** | **#** | **Atom** | **c55** | **C55** | **#** | **Atom** | **c55** | **C55** | **#** | **Atom** | **c55** | **C55** | **#** | **Atom** | **c55** | **C55** |
| --- | --- | --- | --- | --- | --- | --- | --- | --- | --- | --- | --- | --- | --- | --- | --- | --- | --- | --- | --- |
| **1** | *CX1* |  | *0.00* |  |  |  |  |  |  |  |  |  |  |  |  |  |  |  |  |
|  | *HX11* |  | *0.00* |  |  |  |  |  |  |  |  |  |  |  |  |  |  |  |  |
|  | *HX12* |  | *0.00* |  |  |  |  |  |  |  |  |  |  |  |  |  |  |  |  |
|  | *CX2* |  | *0.00* |  |  |  |  |  |  |  |  |  |  |  |  |  |  |  |  |
|  | *CX3* |  | *0.00* |  |  |  |  |  |  |  |  |  |  |  |  |  |  |  |  |
|  | *HX31* |  | *0.00* |  |  |  |  |  |  |  |  |  |  |  |  |  |  |  |  |
|  | *HX32* |  | *0.00* |  |  |  |  |  |  |  |  |  |  |  |  |  |  |  |  |
|  | *HX33* |  | *0.00* |  |  |  |  |  |  |  |  |  |  |  |  |  |  |  |  |
|  | *OXT* |  | *0.00* |  |  |  |  |  |  |  |  |  |  |  |  |  |  |  |  |
|  | C1 | 0.00 | 0.00 | **3** | C1 | 0.00 | 0.00 | **5** | C1 | 0.00 | 0.00 | **7** | C1 | 0.01 | 0.00 | **9** | C1 | 0.00 | 0.01 |
|  | H11 | 0.01 | 0.00 |  | H11 | 0.01 | 0.00 |  | H11 | 0.00 | -0.01 |  | H11 | 0.02 | 0.00 |  | H11 | 0.00 | 0.01 |
|  | H12 | 0.00 | 0.00 |  | H12 | 0.01 | 0.01 |  | H12 | -0.01 | 0.01 |  | H12 | 0.02 | -0.01 |  | H12 | -0.01 | 0.02 |
|  | H13 | 0.00 |  |  |  |  |  |  |  |  |  |  |  |  |  |  |  |  |  |
|  | **C2** | **0.01** | **0.00** |  | **C2** | **0.00** | **0.01** |  | **C2** | **0.00** | **-0.01** |  | **C2** | **0.09** | **-0.01** |  | **C2** | **-0.01** | **0.08** |
|  | H2 | 0.00 | 0.00 |  | H2 | 0.01 | 0.01 |  | H2 | 0.00 | -0.01 |  | H2 | 0.03 | 0.00 |  | H2 | 0.00 | 0.02 |
|  | **C3** | **0.00** | **0.00** |  | **C3** | **0.01** | **0.00** |  | **C3** | **0.00** | **0.01** |  | **C3** | **0.07** | **0.00** |  | **C3** | **0.01** | **0.07** |
|  | C4 | 0.01 | 0.00 |  | C4 | 0.00 | 0.00 |  | C4 | 0.00 | 0.00 |  | C4 | 0.02 | 0.00 |  | C4 | 0.00 | 0.01 |
|  | H41 | 0.01 | 0.00 |  | H41 | 0.00 | 0.00 |  | H41 | 0.00 | 0.01 |  | H41 | 0.02 | 0.01 |  | H41 | 0.01 | 0.03 |
|  | H42 | 0.02 | 0.00 |  | H42 | 0.01 | -0.01 |  | H42 | 0.01 | 0.01 |  | H42 | 0.03 | 0.00 |  | H42 | 0.01 | 0.01 |
|  | H43 | 0.01 | 0.00 |  | H43 | 0.00 | 0.00 |  | H43 | 0.00 | -0.01 |  | H43 | 0.02 | 0.00 |  | H43 | 0.00 | 0.02 |
|  | C5 | 0.00 | 0.00 |  | C5 | 0.00 | 0.00 |  | C5 | 0.00 | 0.00 |  | C5 | 0.02 | 0.00 |  | C5 | 0.00 | 0.01 |
|  | H51 | 0.01 | 0.00 |  | H51 | 0.01 | 0.00 |  | H51 | 0.01 | 0.00 |  | H51 | 0.02 | 0.01 |  | H51 | 0.01 | 0.02 |
|  | H52 | 0.01 | 0.00 |  | H52 | 0.01 | 0.00 |  | H52 | 0.00 | 0.01 |  | H52 | 0.06 | 0.01 |  | H52 | 0.00 | 0.02 |
| **2** | C1 | 0.00 | 0.00 | **4** | C1 | 0.00 | 0.00 | **6** | C1 | 0.00 | 0.00 | **8** | C1 | 0.02 | 0.00 | **10** | C1 | 0.00 | 0.01 |
|  | H11 | 0.01 | 0.00 |  | H11 | -0.01 | 0.00 |  | H11 | 0.01 | 0.00 |  | H11 | 0.01 | -0.01 |  | H11 | 0.00 | 0.00 |
|  | H12 | 0.00 | 0.00 |  | H12 | 0.00 | 0.00 |  | H12 | 0.00 | 0.01 |  | H12 | 0.03 | 0.01 |  | H12 | 0.00 | 0.02 |
|  | **C2** | **0.01** | **0.01** |  | **C2** | **0.00** | **0.00** |  | **C2** | **0.02** | **0.00** |  | **C2** | **0.00** | **0.02** |  | **C2** | **0.00** | **0.01** |
|  | H2 | 0.01 | 0.00 |  | H2 | 0.00 | 0.00 |  | H2 | 0.01 | 0.00 |  | H2 | 0.01 | 0.02 |  | H2 | 0.00 | 0.02 |
|  | **C3** | **0.00** | **0.00** |  | **C3** | **0.01** | **0.00** |  | **C3** | **-0.01** | **0.00** |  | **C3** | **0.02** | **0.00** |  | **C3** | **0.00** | **0.01** |
|  | C4 | 0.00 | 0.00 |  | C4 | 0.00 | 0.00 |  | C4 | 0.01 | 0.00 |  | C4 | 0.01 | 0.00 |  | C4 | 0.00 | 0.01 |
|  | H41 | 0.00 | 0.00 |  | H41 | 0.00 | 0.00 |  | H41 | 0.01 | 0.00 |  | H41 | 0.01 | 0.01 |  | H41 | 0.00 | 0.00 |
|  | H42 | 0.01 | 0.00 |  | H42 | 0.00 | 0.01 |  | H42 | 0.01 | 0.00 |  | H42 | 0.01 | 0.00 |  | H42 | 0.01 | 0.01 |
|  | H43 | 0.00 | 0.00 |  | H43 | 0.01 | 0.00 |  | H43 | 0.01 | -0.01 |  | H43 | 0.01 | 0.00 |  | H43 | 0.00 | 0.01 |
|  | C5 | 0.01 | 0.00 |  | C5 | 0.00 | 0.00 |  | C5 | 0.01 | 0.00 |  | C5 | 0.00 | 0.01 |  | C5 | 0.00 | 0.01 |
|  | H51 | 0.02 | -0.01 |  | H51 | 0.01 | 0.01 |  | H51 | 0.01 | 0.01 |  | H51 | 0.00 | 0.00 |  | H51 | 0.00 | 0.02 |
|  | H52 | 0.01 | 0.00 |  | H52 | 0.00 | 0.01 |  | H52 | 0.01 | 0.00 |  | H52 | 0.01 | 0.00 |  | H52 | 0.00 | 0.01 |
|  |  |  |  |  |  |  |  |  |  |  |  |  |  |  |  |  | *H53* | *0.00* |  |
|  |  |  |  |  |  |  |  |  |  |  |  |  |  |  |  |  | *C6* |  | *0.02* |
|  |  |  |  |  |  |  |  |  |  |  |  |  |  |  |  |  | *H61* |  | *0.01* |
|  |  |  |  |  |  |  |  |  |  |  |  |  |  |  |  |  | *H62* |  | *0.03* |
|  |  |  |  |  |  |  |  |  |  |  |  |  |  |  |  |  | *C7* |  | *0.13* |
|  |  |  |  |  |  |  |  |  |  |  |  |  |  |  |  |  | *H7* |  | *0.06* |
|  |  |  |  |  |  |  |  |  |  |  |  |  |  |  |  |  | *OXT* |  | *0.11* |
